# The Pathophysiology of Portal Vein Thrombosis in Cirrhosis: Getting Deeper into Virchow’s Triad

**DOI:** 10.3390/jcm11030800

**Published:** 2022-02-02

**Authors:** Aina Anton, Genís Campreciós, Valeria Pérez-Campuzano, Lara Orts, Joan Carles García-Pagán, Virginia Hernández-Gea

**Affiliations:** 1 Barcelona Hepatic Hemodynamic Laboratory, Liver Unit, Hospital Clínic, Health Care Provider of the European Reference Network on Rare Liver Disorders (ERN-Liver), 08036 Barcelona, Spain; anton@clinic.cat (A.A.); gcamprecios@clinic.cat (G.C.); vperez2@clinic.cat (V.P.-C.); lorts@clinic.cat (L.O.); JCGARCIA@clinic.cat (J.C.G.-P.); 2 Institut d’Investigacions Biomèdiques August Pi i Sunyer (IDIBAPS), 08036 Barcelona, Spain; 3 Centro de Investigación Biomédica Red de Enfermedades Hepáticas y Digestivas (CIBEREHD), 28029 Madrid, Spain; 4 Medicine Department, Faculty of Medicine, Universitat de Barcelona, 08036 Barcelona, Spain

**Keywords:** portal vein thrombosis, cirrhosis, Virchow’s triad, coagulability, endothelium, blood flow

## Abstract

Portal vein thrombosis (PVT) is a common complication among patients with cirrhosis. However, its pathophysiology is not well established and there are currently very few predictive factors, none of which are actually useful, from a clinical perspective. The contribution of each of the vertices of Virchow’s triad, e.g., blood hypercoagulability, blood flow, and portal vein endothelial damage in the development of PVT is not clear. In this review, we aim to recapitulate the latest studies on the field of PVT development in order to understand its mechanisms and discuss some of the future directions in the study of this important complication of cirrhosis.

## 1. Introduction

Portal vein thrombosis (PVT) is defined as the appearance of a thrombus in the portal vein or its branches, with or without an extension to the superior mesenteric vein or the splenic vein (the so-called splanchnic territory) [1]. PVT is generally asymptomatic and is frequently detected during screening imaging, although in some patients it may cause abdominal pain due to mesenteric ischemia. It may also be discovered during the admission due to portal hypertension-related complications, such as esophageal variceal bleeding [2]. Once detected, a contrast-enhanced CT-Scan/MRI should be performed, with the final aim of evaluating the extension, the guide management, and the evolution during the follow-up and treatment responses. The most recommended characteristics to evaluate are: the initial site, the number of vessels affected, the degree of luminal obstruction, and the time course (acute or chronic) [3].

Specific features of the splanchnic territory in cirrhosis may be responsible for the increased risk of thrombosis in the portal system, over systemic venous circulation. Indeed, the portal venous system is unique. It is responsible for draining the blood from the gastrointestinal tract to the liver and, therefore, it does not drain into the heart but into a capillary system (the hepatic sinusoids) that becomes highly resistant in the setting of cirrhosis. It does not contain valves and it does not have any pulsatile flow that is influenced by the cardiac cycle [4]. In healthy conditions, the portal venous system represents a distinctive vascular environment due to its low-pressure, slow flow, and high-volume hemodynamics. The portal vein compliance is very high (the vessel can be easily distended to compensate variations in portal vein flow), making thrombosis a rare event in the general population (it has a global prevalence of 1%, with an incidence of 0.35–2.5 cases per 100,000 per year, normally in patients with a severe underlying prothrombotic condition) [5]. However, in patients with cirrhosis, even in the absence of thrombophilia, the most frequent site of thrombosis is the splanchnic territory [6], whereas in the general population, thrombosis typically occurs in the lower limbs and, to a lesser extent, in the pulmonary circulation [5], suggesting that the changes that occur in the splanchnic territory during cirrhosis may play a key role in PVT development. Although the prevalence of PVT in cirrhosis varies widely among studies, it is well accepted that up to 10–25% of cirrhotic patients will eventually develop PVT, with its incidence increasing in parallel with the severity of the disease [7,8].

Currently, it is not clear whether PVT is cause or consequence of worsening liver disease, and, therefore, the extent to which PVT influences outcomes in patients with cirrhosis is still a matter of debate. While some studies associate PVT development with further increases in portal hypertension, hepatic decompensation, or even an increased risk of mortality [9], other studies claim that there is no correlation between the development of PVT and the progression of the disease [10,11]. However, data are clearer in the setting of a liver transplantation, where PVT can have a detrimental impact, especially if it is occlusive and it impairs physiological anastomosis [12,13]. The presence of PVT may even be a contraindication for liver transplantation, due to the increased surgical complexity and its association with worse outcomes and increased mortality after the transplant [14,15]. Similarly, the development of post-transplant PVT is also associated with increased morbidity and mortality [16].

Spontaneous PVT recanalization is rare in non-cirrhotic patients but it occurs relatively frequently in patients with cirrhosis [11,17]. Nonetheless, although the spontaneous resolution of PVT may occur, especially if the thrombus is non-occlusive or in the setting of compensated cirrhosis, PVT may also progress, leading to a total occlusion or the reappearance after resolution, suggesting that there are special conditions in the portal territory facilitating PVT development [10,18].

Nowadays, the indications for PVT treatment are not well-defined, and treatment recommendations are only based on expert opinions and consensus [19,20,21]. In the absence of specific data, PVT is currently managed as any other venous thrombosis, with anticoagulation (mostly with low-molecular-weight heparin, vitamin K antagonists, and more recently, direct oral anticoagulants (DOACs)) but, contrary to what happens in other venous territories, the response to anticoagulant therapy in PVT is poor [22,23]. Thirty to 60% of patients with cirrhosis and PVT do not achieve the resolution of the clot and/or the recanalization of the portal vein, despite long-term anticoagulation, therefore, suggesting that the composition of the portal vein thrombus may somehow differ from the ones in the systemic veins [2].

Understanding the role that different factors play in the pathophysiology of PVT seems crucial in order to unveil new putative therapeutic targets that can help to prevent the development of thrombi in the portal vein circulation, or that can achieve recanalization once the thrombus is formed with personalized treatment. The pathogenesis of a thrombus is generally multifactorial and it is believed that its occurrence is mainly determined by the interrelation of the three physiological factors of Virchow’s triad: the hypercoagulability of the blood, the stasis of blood flow, and intravascular vessel wall damage [24,25]. Nevertheless, the exact contribution of each of these factors to PVT development has not been fully elucidated [26,27].

In this review, we aim to incorporate the latest studies on PVT development in the context of cirrhosis with the existent knowledge, as well as discuss all three vertices of Virchow’s triad in detail and propose future directions for the advancement of this field. Both the clinical management of PVT and the invasion of the portal vein due to HCC, which have been reviewed elsewhere [27,28], are out of the scope of this review.

## 2. Blood Hypercoagulability

Liver dysfunction leads to the decreased synthesis of several hemostatic proteins, procoagulants (prothrombin, factor V, and X) and anticoagulant factors (protein C, protein S, and antithrombin), resulting in a reduction in the plasma levels of most of these coagulation pathway components, generating a rebalance of the hemostatic system in patients with cirrhosis [29]. Importantly, this rebalanced equilibrium between pro- and anti-coagulant factors is fragile and represents a real challenge for practitioners. These patients are at a high risk of bleeding due to portal hypertension (e.g., intestinal bleeding, punctures, etc.) but also present with an increased thrombotic risk [29]. PVT is the most common thrombotic complication in these patients [30]. Therefore, being able to assess and stratify each patient according to their risk to either bleed, or develop thrombi, will definitely impact clinical practices and improve patient prognoses. In this regard, many studies have tried to assess the specific role of blood hypercoagulability in the formation of PVT in cirrhotic patients, while many others have searched for the blood biomarkers that are predictive of de novo PVT development.

The standard parameters used to analyze the coagulation status in the general population (prothrombin time, INR, and partial thromboplastin time) are not able to capture the coagulation state of cirrhotic patients. The ratio between FVIII (procoagulant) and protein C (anticoagulant) has long been suggested to reflect the coagulation status as it increases with disease progression. Nonetheless, contradictory results have been published when analyzing this ratio in cirrhotic patients with, vs without, PVT [30,31,32,33]. Recent data demonstrates that this ratio is a versatile predictor of the development of complications of cirrhosis but is unrelated to coagulation [34]. Similarly, the ratio of factor II to protein C has also been shown to decrease in cirrhotic patients with PVT [30,31,33] but its role as an independent risk factor for PVT development has not been demonstrated.

Several studies have depicted changes in coagulation factors when comparing cirrhotic patients that developed PVT versus those that did not (protein C, protein S, antithrombin, soluble F1+2, and factor VIIa); however, none of them are able to predict PVT development [35,36].

La Mura et al. (retrospective, *n* = 53 (11 PVT)) reported thrombomodulin resistance as an independent risk factor for de novo PVT development in cirrhotic patients (HR: 8.354), which was associated with a severe outcome [37], but these data have not been validated in independent cohorts. Ren et al. (retrospective, *n* = 175 (99 PVT)) described a list of altered molecules and factors in cirrhotic patients with PVT, where the most significant ones were the increase in the thrombin–antithrombin complex (TAT) in PVT patients (ROC: 0.68) and the increase in the TAT/t-PAIC (tissue plasminogen activator inhibitor complex) ratio in patients without PVT (ROC: 0.66) [33], which, together with lower levels of vWF:Ag (ROC: 0.61), were proposed as potential biomarkers for predicting PVT occurrence. However, the use of protein C, protein S, antithrombin, FVIII, and the fibrinolytic system PIC (plasmin-alfa2-plasmin inhibitor complex), did not show any capacity to discriminate between patients with and without PVT.

Fimognari et al. (136 consecutive patients (33 PVT)) explored the use of the coagulation and fibrinolysis markers, D-dimer and FVIII, as predictive factors for PVT development in cirrhotic patients. They observed, however, that factor VIII increases in severe cirrhosis but significantly decreases in the presence of concomitant portal thrombosis, while D-dimer is enhanced by both liver dysfunction and PVT, although their discriminative ability was unsatisfactory [38].

Taking a different approach to try and determine the blood hypercoagulability status of cirrhotic patients with or without PVT, Rossetto et al. used rotational thromboelastometry (ROTEM). ROTEM investigates the interaction of coagulation factors, their inhibitors, and blood cells, specifically platelets, during the clotting and subsequent fibrinolysis. It is able to detect both the hypo- and hyperfunctional stages of the clotting process. In this context, however, ROTEM could only differentiate patients with cirrhosis from controls, but none of the parameters were statistically significant between cirrhotic patients with and without PVT [39].

Overall, the above studies are quite inconclusive regarding the role of blood hypercoagulability in the development of PVT in cirrhotic patients. Despite studies reporting differences between patients with or without PVT, the levels of most of these pro- and anti-coagulant factors are generally not statistically significant, especially when correcting for the disease stage. Moreover, many of these studies included a low number of patients, are mostly retrospective, and are without a follow-up, which occasionally leads to contradictory results.

In the largest prospective study published so far (369 patients) and with a long follow-up (up to 48 months), performing an exhaustive evaluation of clinical, biochemical, inflammatory, acquired, and hereditary hemostatic profiles [31], as well as alterations in the hemostatic parameters, did not predict de novo PVT development. Regarding the biochemical traits, the study confirmed that many markers beyond the FVIII to protein C ratio, thrombomodulin resistance and the generation and vWF were altered, but none of them were independently associated with PVT development during the follow-up related to the stage of liver disease. These results are in agreement with other recently published studies [11,40], together leading to the conclusion that blood hypercoagulability may not be the culprit of PVT development.

Additionally, the prevalence of the underlying acquired (myeloproliferative neoplasms, polycythemia vera), or inherited (protein C and protein S deficiencies, mutations in prothrombin (G20210A), factor V Leiden, etc.), prothrombotic diseases is very low in patients with cirrhosis [26,41], further suggesting the non-predominant role of hypercoagulability in PVT development.

All the studies described above evaluated coagulation factors in systemic blood but its representative value to extrapolate what happens in the portal vein is uncertain. Delahouse et al. [42] and Praktiknjo et al. [43] evaluated hypercoagulability in the splanchnic territory when compared to systemic circulation. Although both studies described the existence of relative hypercoagulability in the portal vein of cirrhotic patients (a higher concentration of vWF and factor VIII, as well as decreased protein C and protein S), none of the studies evaluated patients with PVT, and, therefore, the contribution of this gradient remains unclear.

Finally, another important factor influencing blood hypercoagulability is inflammation. The role of cirrhosis-associated inflammation in PVT development has been recently analyzed by quantifying neutrophil extracellular traps (NETs), but no association could be demonstrated [31], although the study did not analyze portal vein blood. A study by Huang et al. (retrospective, *n* = 231 (103 PVT)), however, pointed to systemic inflammation as a possible factor for PVT development [44]. Similar to this, several studies have shown that a higher percentage of NASH patients awaiting transplant develop PVT, compared to other etiologies [45,46]. Since this phenomenon does not seem to be related to blood hypercoagulability [47,48], it is plausible that the liver microenvironment of these patients, with manifested systemic inflammation, might be actively contributing to PVT formation.

In summary, although hypercoagulability is associated with the progression of liver disease and portal hypertension, the available data does not support a major role for blood hypercoagulability in the pathogenesis of PVT.

## 3. Portal Vein Hemodynamics and Blood Flow

Portal vein hemodynamic changes due to portal hypertension play an important role in the development of PVT in cirrhotic patients. During fibrosis accumulation, intrahepatic vascular resistance increases and leads to the appearance of portal hypertension; in turn, portal hypertension induces an increase in the systemic production of vasodilators, as well as a defective systemic response to vasoconstrictors, which eventually leads to splanchnic arteriolar vasodilation, increasing portal vein inflow. As the portal pressure depends (as in any other venous system) on flow and the resistance to the flow (Ohm’s law), the described changes certainly lead to the aggravation and perpetuation of portal hypertension [49]. In the context of high pressure, there is generally an enlargement of the portal vein [50] together with the formation of porto-collateral vessels [51], which divert an important amount of blood from the portal to the systemic circulation, bypassing the liver. It is interesting to note that both a higher portal vein diameter and the presence of large porto-collateral vessels have been associated with an increased risk of developing PVT [10,52,53].

Importantly, the “steal effect” produced by porto-collateral circulation, combined with the increase in portal vein diameter, leads to a reduction of portal blood flow and portal flow velocity. In this regard, in 2009 Zocco et al. published a prospective study of 100 cirrhotic patients followed up after one year and observed that a reduction in the portal vein flow velocity was independently associated with the occurrence of PVT [6]. More precisely, they set a flow velocity below 15 cm/second at baseline as the threshold to discriminate between patients at high and low risk for the de novo appearance of PVT (*p* < 0.001). This result has since been reproduced by other retrospective and prospective studies [31,52,54] and has, therefore, established portal flow velocity as one of the few clear predictive factors for PVT development, independent of cirrhosis severity.

In the last few years, some concerns have been raised regarding the use of nonselective β-blockers (NSBB) in the treatment for portal hypertension, due to their effect on reducing portal blood flow. The use of NSBB in cirrhosis is usually prescribed to those patients with portal hypertension and esophageal varices that prevent either first bleeding or re-bleeding [20]; that is, patients with already high portal hypertension and a high probability of having low portal flow velocity. The NSBB mechanism of action involves the blockade of both β1 and β2 adrenergic receptors, with a consequent decrease in heart rate and cardiac output and the promotion of splanchnic vasoconstriction, altogether reducing portal blood flow and, consequently, portal hypertension. This effect of NSBB on the reduction of portal blood flow led several researchers to try and ascertain whether cirrhotic patients under NSBB may have a higher risk of developing PVT. Although a systemic review and meta-analysis published in 2019, including nine studies, suggested an increased rate of PVT development in patients on NSBB [55], the high heterogeneity of the studies included, most of them retrospective with a short follow-up, and the fact that none of them evaluated the potential role of confounding factors, weakens the results of this observation. The only prospective study performing a time-varying model that considered the changes in NSBB (with a withdrawal or a new prescription during follow up) and adjusted by variceal hemorrhage and the presence of large esophageal varices as longitudinal potential confounders, did not confirm the association of NSBB with an increased risk for PVT. Indeed, this study showed that the severity of portal hypertension (*p* < 0.015) and a portal blood flow velocity of <15 cm/s (*p* < 0.008) were the main risk factors predicting PVT development [31].

Therefore, in the absence of new high-quality data, there is no reason to restrict NSBB prescription in cirrhotic patients based on concerns regarding increasing PVT risk.

## 4. Intravascular Vessel Wall (Endothelial) Damage

The active role of the endothelium in the control of hemostasis is well-known, although its particular involvement in PVT generation has been little studied (partially due to the inaccessibility of the splanchnic territory), and most of our knowledge comes from studies in the systemic venous system (for a review, see [56,57]). The functions attributed to the endothelium, regarding coagulation, can be separated in two main interrelated categories. The first category is its location. Endothelial cells line the inner walls of vessels and are in direct contact with blood. They behave as a barrier, preventing blood clotting factors from being exposed to subendothelial prothrombotic extracellular matrix components. Once the endothelial wall is damaged, the wound-healing response leads to clot formation to stop/prevent blood loss, which resolves over time. However, this may become chronic and result in thrombosis, an event that is frequently seen in the arterial system after the rupture of atherosclerotic plaques [58], but rarely in the venous system. The second category is that the endothelium expresses and secretes a plethora of different factors that regulate the activation of platelets, the coagulation cascade, fibrinolysis, and vascular contractility, processes that contribute to the regulation of hemostasis and thrombi formation. These factors include anti-coagulant molecules such as nitric oxide (NO), prostacyclin, thrombomodulin, the tissue factor pathway inhibitor (TFPI), and protein C receptors, as well as pro-coagulants, such as vWF, tissue factors (TF), P-selectin, FVIII, and endothelin. The net balance between pro- and anticoagulants, which is directly affected by endothelial dysfunction, will ultimately determine the role endothelial cells will play in each specific setting. Under homeostatic conditions, endothelial cells display an anti-coagulant phenotype and help to control adequate blood flow and tissue perfusion; however, under stress conditions, endothelial cells activate (dysfunctionally) and acquire a pro-coagulant phenotype, contributing to both the initiation and the propagation of thrombus generation [59].

In cirrhosis, endothelial dysfunction has been inferred through different studies that show an upregulation of markers such as P-selectin [60,61], vWF [62,63], and isoprostanes in systemic venous blood [64]. Portal hypertension and bacterial translocation are both believed to contribute to the activation of the endothelium. Indeed, Ferlitsch et al. found a correlation between high levels of vWF and clinically significant portal hypertension, decompensation, and mortality [65]. On the other hand, bacteremia, due to bacterial translocation, has been shown to be higher in the portal area, as well as inducing the endothelial expression of vWF and FVIII [66,67].

Along these lines, a recent study by Praktiknjo et al. comparing peripheral and portal venous blood from 20 cirrhotic patients and described increased amounts of vWF in the portal circulation [43], as well as endothelial-secreted FVIII, reinforcing a previous study by Delahousse et al. [42] with 11 subjects, and suggested that there is portal endothelial damage. Supporting this information, Shalaby et al. also showed the presence of endothelial damage in the portal vein by comparing peripheral and portal venous blood from 45 cirrhotic patients [68]. They observed an increase in the amount of circulating sulfated glycosaminoglycans (GAGs), a surrogate marker of endothelial damage, in the portal system, indicating sustained damage to the portal endothelial glycocalyx. Additionally, they also detected a slightly higher percentage of endothelial-derived microparticles (MP, annexin V+, and CD62+) in the portal area, compared to the systemic venous system. Microparticles, which are small membrane fragments with procoagulant properties, are constitutively released from the surface of blood and endothelial cells and their formation is upregulated during cellular activation and apoptosis [69]. In this regard, two other studies found increased levels of thrombomodulin and t-PAIC, two other markers previously used to infer endothelial cell injury [70,71].

Therefore, a higher presence of these endothelial-derived MPs in the portal area, together with the increased levels of GAGs, vWF, and FVIII, all support the existence of the endothelial damage of the portal vein in patients with cirrhosis.

In summary, to better ascertain the possible implications of the endothelium in the development of PVT in cirrhosis, more studies are needed that compare endothelial-specific markers in blood from the portal area between cirrhotic patients with or without PVT.

## 5. Thrombus Composition

Today, PVT is still a challenging and quite enigmatic entity. Besides the complexity of its formation, which has been extensively discussed in the above sections, there are two additional interlinked aspects that make PVT a unique entity among venous thromboses. Natural history studies have described PVT as a transient entity that can recanalize during follow-up, even in the absence of treatment. However, intriguingly enough, the rates of portal vein recanalization after anticoagulation are substantially lower, especially in an aged thrombus.

Both of these observations are hard to explain with the current approaches and deserve further investigation. In this direction, the recently published study by Driever et al. [72] challenges the current view of PVT and its pathophysiology. In their study, they analyzed 16 prospectively and 64 retrospectively collected portal vein segments from cirrhotic patients, in whom there was supposed to be a thrombus, and vastly analyzed both the vessels and thrombi using histology and electron microscopy. They concluded that in all of those specimens, rather than just PVT, the occlusion of the portal vein lumen was mostly due to a thickening of the tunica intima in an appearance that resembles intimal fibrosis. Additionally, in approximately one-third of those cases, they also found a fibrinogen-rich blood thrombus, which was distinct to those described in deep vein thrombosis or arterial clots [72,73]. These results led them to propose new terminology that reflected their main findings, such as “portal vein stenosis” or “non-malignant portal vein occlusion” and even led them to speculate that the low rate of recanalization observed in PVT cirrhotic patients, with the use of anticoagulants, might be due to the nature of this portal vein occlusion, suggesting that recanalization might only be accomplished in those cases where fibrin-rich clots are present.

This novel and intriguing finding defies the current understanding of PVT pathophysiology and may have important clinical implications if validated in future studies. Undoubtedly, these data encourage future studies aimed to better understand PVT development and to shed light on possible new treatment interventions.

## 6. Main Challenges and Future Directions

Studies that uncover PVT pathophysiology have been hampered by two main issues. On one hand, the portal vein area has historically been, and it is still today, a territory difficult to access. On the other hand, there is a lack of preclinical models for the study of thrombus generation and the evaluation of therapies; the ones that currently exist require either initial artificial direct damage to the venous wall, a partial ligation of the vessel, or both [74] and, therefore, do not recapitulate the complexity of the cirrhosis-associated changes in the splanchnic vascular bed. In consequence, most of the accumulated knowledge on PVT gathered in this review comes from clinical association studies between systemic blood parameters and clinical information.

**Figure 1:**
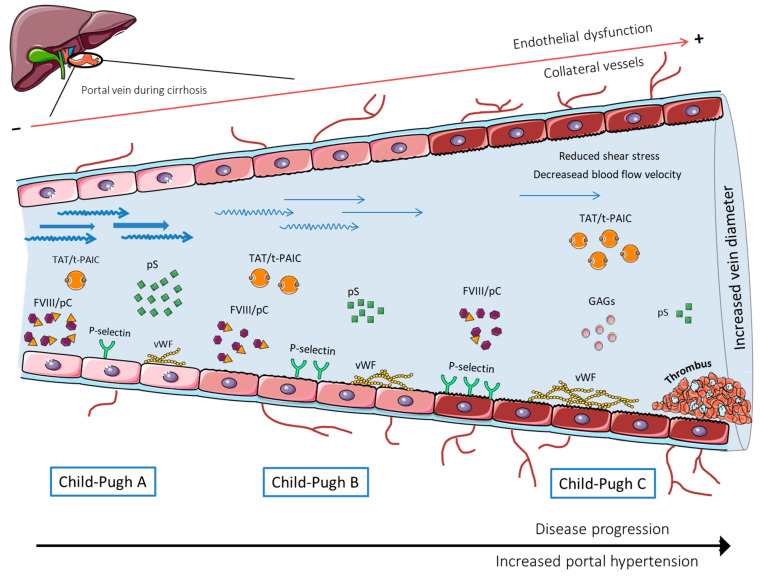
Scheme of the changes occurring in the portal vein during the different cirrhotic stages.

Nonetheless, and coming back to Virchow’s triad, the results discussed above reflect the complexity of PVT in the context of cirrhosis, where alterations in all the different components of the triad coexist and worsen as the disease progresses and as portal hypertension increases, most likely all contributing, to some extent, to the formation of PVT (Figure 1). This parallelism, in turn, is what makes it difficult to detect factors that stand alone in a multivariate analysis where disease staging is also incorporated.

That said, the portal vein flow velocity and specific endothelial factors have shown an association with de novo PVT development in some prospective studies, while blood coagulability seems to be an accompanying confounding factor. However, one could also argue that portal vein flow velocity is, in turn, a passive passenger of endothelial dysfunction. Cirrhosis and portal hypertension are the result of a liver disease that initially started by an endothelial dysfunction of the liver sinusoids, leading to an increase in intrahepatic vascular resistance that caused portal hypertension, which then created all the alterations in the systemic and splanchnic circulations, perpetuating and worsening this condition and, as a consequence, slowing the portal blood flow velocity.

Alternatively, the thickening of the tunica intima of the vessel would still be the result of initial endothelial stress produced in the portal endothelium due to the harsh conditions that coexist in that territory during cirrhosis development, which would lead to vascular damage, platelet aggregation, leukocyte chemotaxis, and endothelial proliferation, ultimately resulting in the proliferation of mesenchymal cells and deposition of extracellular matrix components in the tunica intima.

In any case, while many studies have evaluated the dysfunction of the liver endothelium during fibrosis and cirrhosis progression, not much research has been dedicated to the portal vein endothelium, which the current data indicates might play an active role in PVT formation, both directly and indirectly. Thus, deciphering which mechanisms govern this endothelial dysfunction and predisposes it to favor PVT development could be of great interest to uncover new therapeutic targets that help in both the prevention and the recanalization of those thrombi that do not respond to classical anticoagulant drugs. In the meantime, however, anticoagulants will remain the therapeutic choice for cirrhotic patients with PVT.
